# Advanced Warning of Aortic Dissection on Non-Contrast CT: The Combination of Deep Learning and Morphological Characteristics

**DOI:** 10.3389/fcvm.2021.762958

**Published:** 2022-01-05

**Authors:** Yan Yi, Li Mao, Cheng Wang, Yubo Guo, Xiao Luo, Donggang Jia, Yi Lei, Judong Pan, Jiayue Li, Shufang Li, Xiu-Li Li, Zhengyu Jin, Yining Wang

**Affiliations:** ^1^Department of Radiology, State Key Laboratory of Complex Severe and Rare Diseases, Peking Union Medical College Hospital, Chinese Academy of Medical Science and Peking Union Medical College, Beijing, China; ^2^AI Lab, Deepwise Healthcare, Beijing, China; ^3^Department of Radiology, The First Affiliated Hospital of Shenzhen University, Health Science Center, Shenzhen Second People's Hospital, Shenzhen, China; ^4^Department of Radiology and Biomedical Imaging, University of California San Francisco (UCSF), San Francisco, CA, United States; ^5^School of Computing, Informatics, and Decision Systems Engineering, Arizona State University, Tempe, AZ, United States; ^6^School of Information and Communication Engineering, Beijing University of Posts and Telecommunications, Beijing, China

**Keywords:** aortic dissection, computed tomography angiography, diagnostic imaging, multidetector computed tomography, deep learning

## Abstract

**Background:** The identification of aortic dissection (AD) at baseline plays a crucial role in clinical practice. Non-contrast CT scans are widely available, convenient, and easy to perform. However, the detection of AD on non-contrast CT scans by radiologists currently lacks sensitivity and is suboptimal.

**Methods:** A total of 452 patients who underwent aortic CT angiography (CTA) were enrolled retrospectively from two medical centers in China to form the internal cohort (341 patients, 139 patients with AD, 202 patients with non-AD) and the external testing cohort (111 patients, 46 patients with AD, 65 patients with non-AD). The internal cohort was divided into the training cohort (*n* = 238), validation cohort (*n* = 35), and internal testing cohort (*n* = 68). Morphological characteristics were extracted from the aortic segmentation. A deep-integrated model based on the Gaussian Naive Bayes algorithm was built to differentiate AD from non-AD, using the combination of the three-dimensional (3D) deep-learning model score and morphological characteristics. The areas under the receiver operating characteristic curve (AUCs), accuracy, sensitivity, and specificity were used to evaluate the model performance. The proposed model was also compared with the subjective assessment of radiologists.

**Results:** After the combination of all the morphological characteristics, our proposed deep-integrated model significantly outperformed the 3D deep-learning model (AUC: 0.948 vs. 0.803 in the internal testing cohort and 0.969 vs. 0.814 in the external testing cohort, both *p* < 0.05). The accuracy, sensitivity, and specificity of our model reached 0.897, 0.862, and 0.923 in the internal testing cohort and 0.730, 0.978, and 0.554 in the external testing cohort, respectively. The accuracy for AD detection showed no significant difference between our model and the radiologists (*p* > 0.05).

**Conclusion:** The proposed model presented good performance for AD detection on non-contrast CT scans; thus, early diagnosis and prompt treatment would be available.

## Introduction

Aortic dissection (AD) is a life-threatening disease for which early diagnosis and treatment are critical. The mortality rate increases by 1–2% per hour after symptom onset (1). Typically, patients may present with symptoms such as sudden onset of severe chest pain or back pain. To date, CT angiography (CTA) is the best imaging modality for identifying displaced intimal flaps in contrast-enhanced scans, with a sensitivity and specificity approaching 100% (2, 3).

However, CTA is to some degree restricted due to the allergenicity and nephrotoxicity of contrast agents and the lack of 24-h availability in some emergency departments, particularly in rural or underserved areas that lack technical and staff support (4). Moreover, many patients who present atypical or asymptomatic AD in the early stages have been missed diagnosed and deteriorate rapidly (5). In comparison, non-contrast CT scans are widely available, convenient and easy to perform, and have relatively lower radiation doses (6–8). The imaging characteristics of AD on non-contrast CT scans include displaced calcified intimal flaps, intraluminal linear high density, intramural hematoma, and aneurysmal dilatation. However, technical level of the radiologists for AD detection on non-contrast CT scans is suboptimal and currently lacks sensitivity (9).

Compared with traditional methods, the deep learning (DL) algorithms have advantages in the extraction and recognition of subtle differences in digital imaging information. He et al. proposed residual networks (ResNets) (10) that won first place on the ImageNet Large Scale Visual Recognition Challenge (11), which outperformed human accuracy in image classification. Hata et al. (12) designed a DL algorithm for the detection of AD on non-contrast CT; however, their method was limited to two-dimensional (2D) models with image data from a single center.

In this study, we hypothesized that a machine learning model that integrated the prediction of the DL model and morphological characteristics could effectively detect AD on non-contrast CT images. The aim of this study was to build the DL-based model for the early detection of AD using non-contrast CT scans and to demonstrate that the combination of morphological characteristics can strengthen the model performance. We further validated and compared its detection performance with three radiologists at two independent centers.

## Materials and Methods

This study was reviewed and approved by the local clinical Institutional Ethics Committees of the two centers involved and a written informed consent was waived because of the retrospective nature of this study.

### Population of Patient

Between July 2014 and April 2020, 5,885 consecutive patients underwent CTA scans at the Peking Union Medical College Hospital (PUMCH), Beijing, China. The presence of AD was confirmed by the CTA interpretation results and 191 patients were diagnosed with AD. After the inclusion and exclusion criteria were applied (detailed in Supplementary S1), 139 patients with AD were enrolled and 202 patients diagnosed without AD from the same period were approximately propensity matched from the remaining 5,694 patients with non-AD, considering two variables (age and sex).

Thus, 341 patients were enrolled from the PUMCH and were randomly divided into the training cohort (70%, 238 patients with 96 patients with AD and 142 patients with non-AD), validation cohort (10%, 35 patients with 14 patients with AD and 21 patients with non-AD), and internal testing cohort (20%, 68 patients with 29 patients with AD and 39 patients with non-AD).

**Figure 1:**
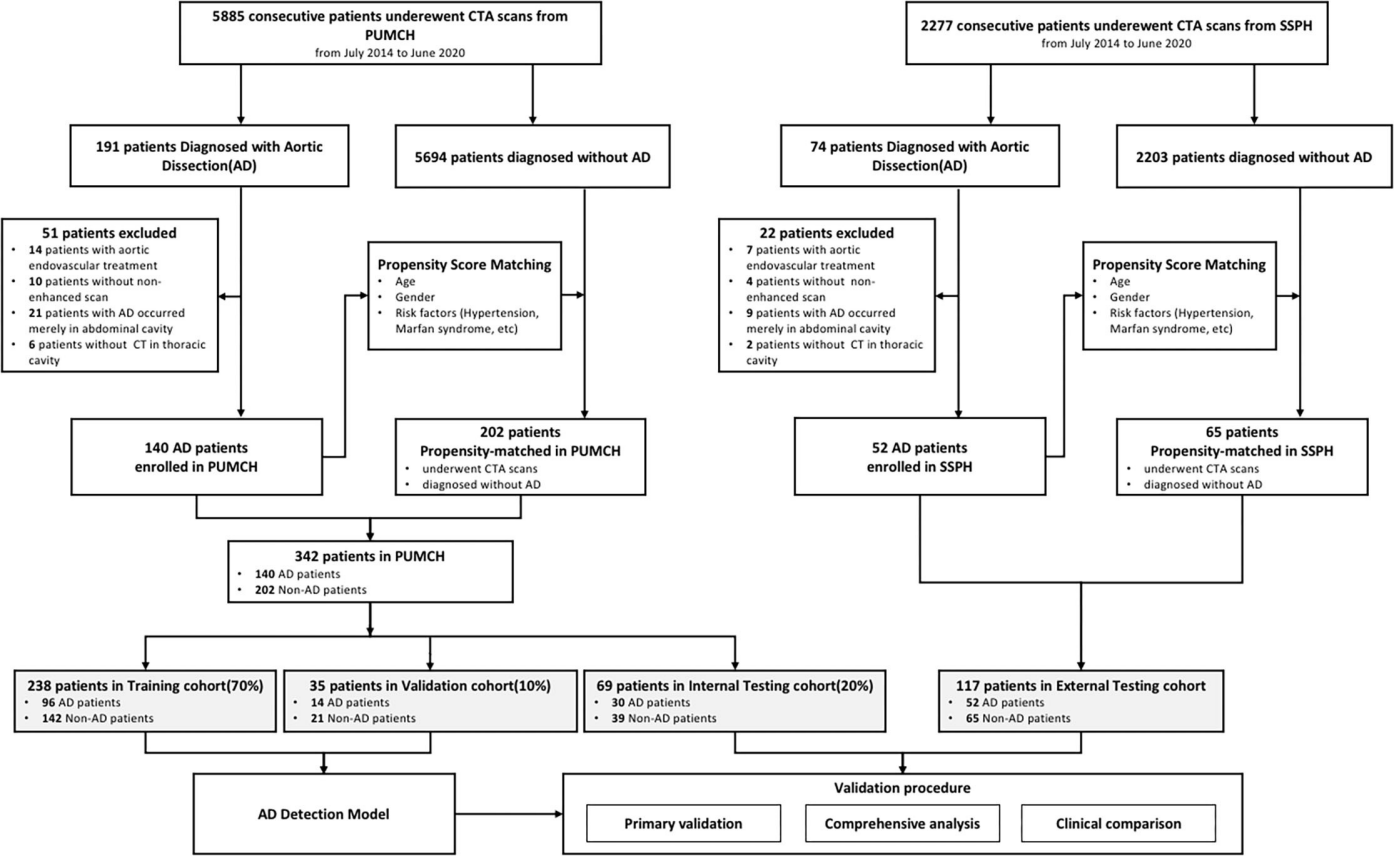
Flowchart of enrollment of patient.

From another independent medical center, the Shenzhen Second People's Hospital (SSPH), Shenzhen, China, 2,273 consecutive patients underwent CTA scans between July 2017 and June 2020. Among them, 70 patients were diagnosed with AD. After the same inclusion and exclusion criteria were applied, 46 patients with AD were enrolled and 65 patients with AD were propensity matched. Then, the external testing cohort was constructed (Figure 1).

The DL model was trained on the training cohort and the validation cohort was used to decide the stopping iteration. The Gaussian Naive Bayes (Gaussian NB) algorithm-based models were trained on the combined training and validation cohorts. After the training procedure, both models were evaluated on the internal testing cohort and external testing cohort.

### Computed Tomography Image Data Acquisition

All the CT scans were performed using post-64-detector row CT scanners from Siemens (Somatom Definition Flash or Somatom Force, Forchheim, Germany) and Philips (iCT Elite FHD or IQon Spectral CT, The Netherlands). Every scan began with non-contrast scanning from the thoracic inlet to the pubic symphysis to cover the entire aorta. Afterward, contrast-enhanced CT scans were performed over the same area during the systemic arterial phase. The slice thickness was 1–5 mm for non-contrast CT images and 1 mm for contrast-enhanced CTA images. The other scanning parameters were as follows: rotation time 0.5 s, pitch 1.2, matrix 512 × 512, standard resolution algorithms, tube voltage 80–100 kV (Somatom Definition Flash, Somatom Force, Forchheim, Germany) and 120 kVp (iCT Elite FHD, IQon Spectral CT, The Netherlands), and the tube current adjusted automatically.

### Radiologists Interpretation of CT Images

The diagnostic interpretations were performed by three radiologists including a junior radiologist with 7 years of experience in cardiovascular imaging [radiologist 1 (YY)] and two senior radiologists with 14 and 16 years of experience [radiologist 2 (ZD) and radiologist 3 (YW), respectively]. These three radiologists interpreted the anonymous non-contrast CT images independently and indicated their dichotomous diagnosis (AD and non-AD).

The characteristics of AD on the non-enhanced CT images included aortic calcification deviation (>5.0 mm), signs of intimal flap, and high-density areas in the aorta; the indirect parameters included uneven density in the aorta, limited or extensive aortic dilatation, irregular aortic morphology, and pericardial or pleural effusions (9, 13, 14).

### Overview of the Model Construction

**Figure 2:**
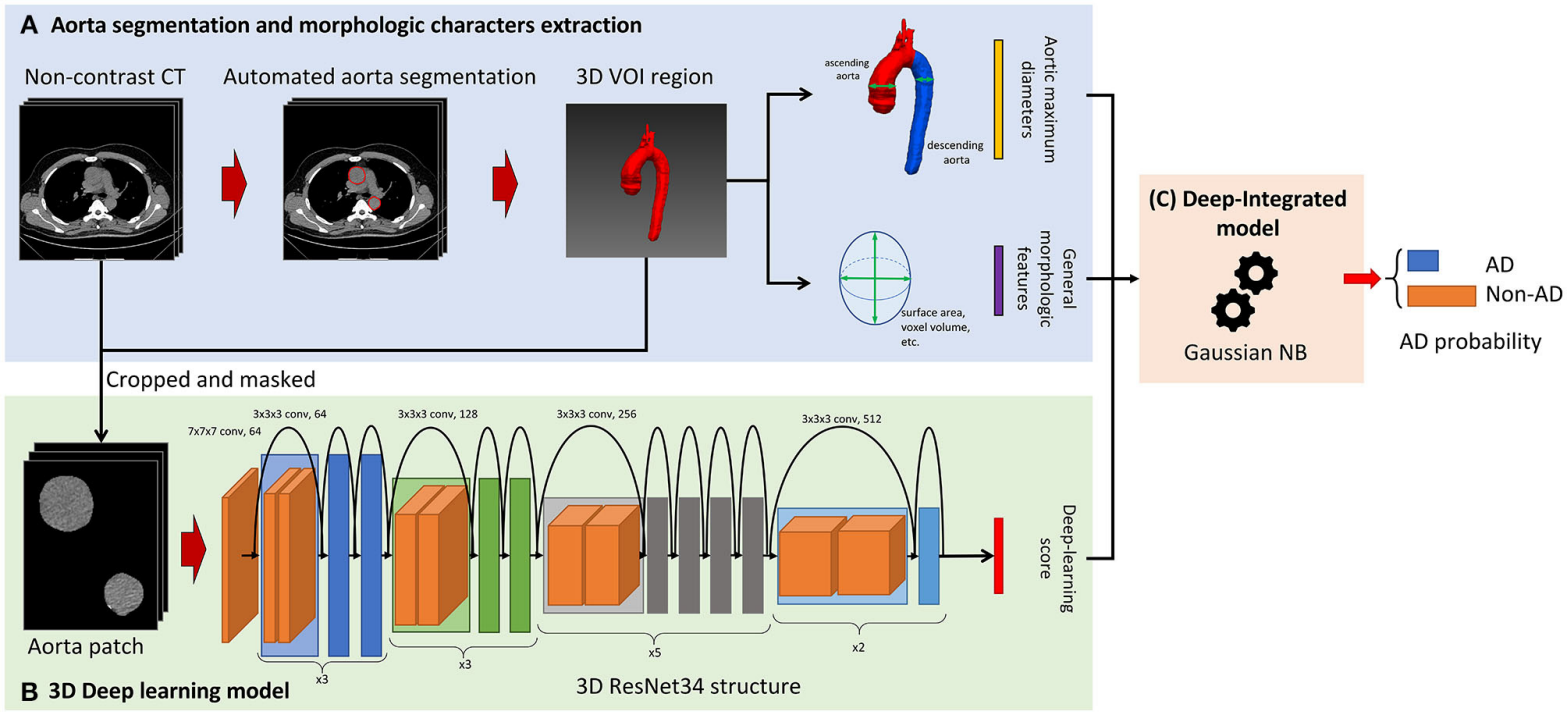
Overview of the model construction for aortic detection. **(A)** The non-contrast CT images were segmented automatically to obtain the aorta mask. Then, the morphological characteristics were extracted from the aorta mask including the aortic maximum diameters and general morphological features. **(B)** The input of the three-dimensional (3D) ResNet34 model was cropped and masked by aorta segmentation and the prediction probability of the deep learning (DL) model was used as the DL score. **(C)** The deep-integrated model was based on the Gaussian Naive Bayes (NB) algorithm and trained on the combination of the DL score and all the morphological characteristics to predict the status of aortic dissection (AD).

An overview of the model construction is given in Figure 2. Before AD detection model building, aorta segmentation was performed to find the three-dimensional (3D) Volume of Interest (VOI) region of the aorta. Then, it was used to crop the aorta volume and extract the morphological characteristics including the aortic maximum diameters and general morphological features. In this study, a 2-stage AD detection model was built. First, as shown in Figure 2B, the 3D DL model based on ResNet34 was built and the prediction probability of the DL model was used as the DL score. Finally, as shown in Figure 2C, our proposed deep-integrated model was based on the Gaussian NB algorithm and trained on the combination of the DL score and all the morphological characteristics to predict the AD status.

### Aorta Segmentation and the Extraction of Morphological Characteristics

The aorta mask was extracted by the 2.5D UNet-based DL model, which was trained and validated on the in-house dataset and is given in Supplementary S2. Morphological characteristics were extracted from the aorta mask including the aortic maximum diameters [the maximum diameter of the ascending aorta (AC) and the maximum diameter of the descending aorta (DC)] and 14 general morphological features extracted by PyRadiomics (version 3.0). The aortic maximum diameters were binarized by the threshold of 4 and 5 cm to form four aortic maximum diameter features, i.e., AC > 4 cm (1 for AC > 4 cm and 0 for AC ≤ 4 cm), AC > 5 cm (1 for AC > 5 cm and 0 for AC ≤ 5 cm), DC > 4 cm (1 for DC > 4 cm and 0 for DC ≤ 4 cm), and DC > 5 cm (1 for DC > 5 cm and 0 for DC ≤ 5 cm). The general morphological features were normalized by z-score normalization. The morphological characteristics are given in Supplementary S3.

### Three-Dimensional Deep-Learning Model for AD Classification

After aorta segmentation, the aorta mask was used to crop the aorta volume. Only aorta pixel values were kept in the aorta volume and then the aorta volume was resized to 64 × 64 × 64. The values were truncated to the mediastinum window (50, 350) and the volumes were employed as the input of the 3D DL model.

We used 3D ResNet (15) as a basic structure of the detection model. 3D ResNet combined an encoder with a fully connected layer for classification (classifier). The same modification of the encoder as MedicalNet (15) was adopted to perform transfer learning using the pre-trained weight from 23 public medical datasets. The optimization was performed by binary cross-entropy loss with the Stochastic Gradient Descent (SGD) optimizer with learning rates of 0.001 and 0.01 for the encoder and classifier, respectively. The weight decay of the SGD optimizer was 0.001 and the momentum was 0.9.

After the 3D DL model was built, the prediction probability of the existence of AD was used as the DL score (Figure 2B). The higher the DL score is, the more likely that the 3D DL model indicates the existence of AD.

### Proposed Model Combined With the DL Score and Morphological Characteristics

Based on the previously calculated morphological characteristics (the aortic maximum diameters and general morphological features) and the DL score, a model based on the Gaussian NB algorithm was built to predict the AD status (deep-integrated model). The deep-integrated model was built on the basis of the 3D DL model; thus, it integrated the 3D information. The optimal subset of morphological characteristics was selected by the Spearman's rank correlation test and the characteristics with a *p*-value < 0.05/18 (Bonferroni correction, 18 tested features) was remained.

The deep-integrated model was trained based on the Gaussian NB algorithm and for the Gaussian NB algorithm for classification, the likelihood of the features is assumed to be Gaussian:


$$\begin{array}{l} P\left(x_{i}|y\right)=\frac{1}{\sqrt{2\pi }\sigma_{y}}\exp\left(-\frac{{\left({\text{x}}_{\text{i}}-\mu_{y}\right)}^{2}}{2\sigma_{y}^{2}}\right) \end{array}$$

The parameters μ and σ are estimated using maximum likelihood.

The training cohort and the validation cohort were merged to train the deep-integrated model using the 10-fold cross-validation procedure. For each iteration of the cross-validation, the model was trained 9-fold and validated on the remaining 1-fold. Then, the validation folds were assembled to form the cross-validation result. After the optimal hyperparameters were selected by the cross-validation result, the final integrated model was retrained on the merged cohort using the optimal hyperparameters and the performance on the internal and external testing cohorts was evaluated by quantifying the accuracy, sensitivity, specificity, and the area under the receiver operating characteristic curve (AUC) (Figure 2C).

### Comprehensive Comparison of the Deep-Integrated Model

To further validate the effectiveness of each feature, based on the aortic maximum diameters and DL scores, the deep maximum diameters model (deep MD model) was built on the basis of the Gaussian NB algorithm by the same procedure as the integrated model and a comparison of the 3D DL model, deep MD model, and deep-integrated model was performed.

The performance of the integrated model on different subtypes was compared. The internal testing cohort and the external testing cohort were divided by the Stanford type diagnosed by the CTA scans and the accuracy of the model and radiologist on these subsets were evaluated and compared.

The robustness of the deep-integrated model at different slice thicknesses was further evaluated. Among the 111 patients in the external testing cohort (SSPH), 63 patients only underwent non-contrast scans with a slice thickness > 8 mm. However, the slice thickness of the training cohort and internal testing cohort from PUMCH was <5 mm. It is important to monitor the impact of performance based on the slice thickness. The external testing cohort was divided according to whether the scans were thicker than 8 mm and the performance was compared.

### Statistical Analysis

All the statistical results were calculated in Python and R (version 3.6.0; https://www.r-project.org/) environments. The demographics of the patient among the three cohorts were compared by the ANOVA tests or the Pearson's chi-squared test when appropriate. For the AD detection model and radiologist assessment, we used the Pearson's chi-squared test with the Yates' continuity correction to compare the sensitivity, specificity, negative predictive value (NPV), and positive predictive value (PPV) of the classification model and artificial interpretation of non-enhanced CT scans. The Fleiss's kappa coefficient was used to measure the consistency of the 3 radiologists. The AUC (0.95 CI) was calculated to evaluate model performance in the two data centers. The two AUCs were compared by the DeLong method (16). A *p*-value of < 0.05 was considered to indicate a significant difference.

## Results

### Population of Patient

In total, 452 patients were enrolled and divided into the training cohort (*n* = 238), validation cohort (*n* = 35), internal testing cohort (*n* = 68), and external testing cohort (*n* = 111). Table 1 shows the detailed demographics of patient and CT image parameters of the training, validation, internal testing, and external testing cohorts. The training cohort and the validation cohort were combined because they were used to find the optimal hyperparameters and to train the model. There were no significant differences in terms of age among the cohorts (*p* = 0.845), but sex was significantly different (*p* = 0.002). It should be noted that the slice thickness was significantly different (*p* < 0.001) and the CT scans in the SSPH cohort were thicker than those in the PUMCH cohort.

**Table 1:** Demographics of patient and CT image parameters in all the cohorts.

	**Training and validation cohorts**	**Internal testing cohort**	**External testing cohort**	***p*-value**
Number of patients	273	69	117	–
**Patients demographics**				
Age, mean ± SD, year	55.47 ± 15.41	55.88 ± 16.64	54.62 ± 17.51	0.851
Sex, No. (%)				0.002
Male	183 (67.03)	37 (54.62)	92 (78.63)	
Female	90 (32.97)	32 (46.38)	25 (21.37)	
**CT image parameters**				
Slice thickness	2.66 ± 2.34	2.72 ± 2.37	7.25 ± 3.43	<0.001
Stanford type				0.401
A	39	13	21	
B	71	16	25	

*The p-values were calculated by the ANOVA test or the Pearson's chi-squared test when appropriate*.

### Performance of the Models

**Figure 3:**
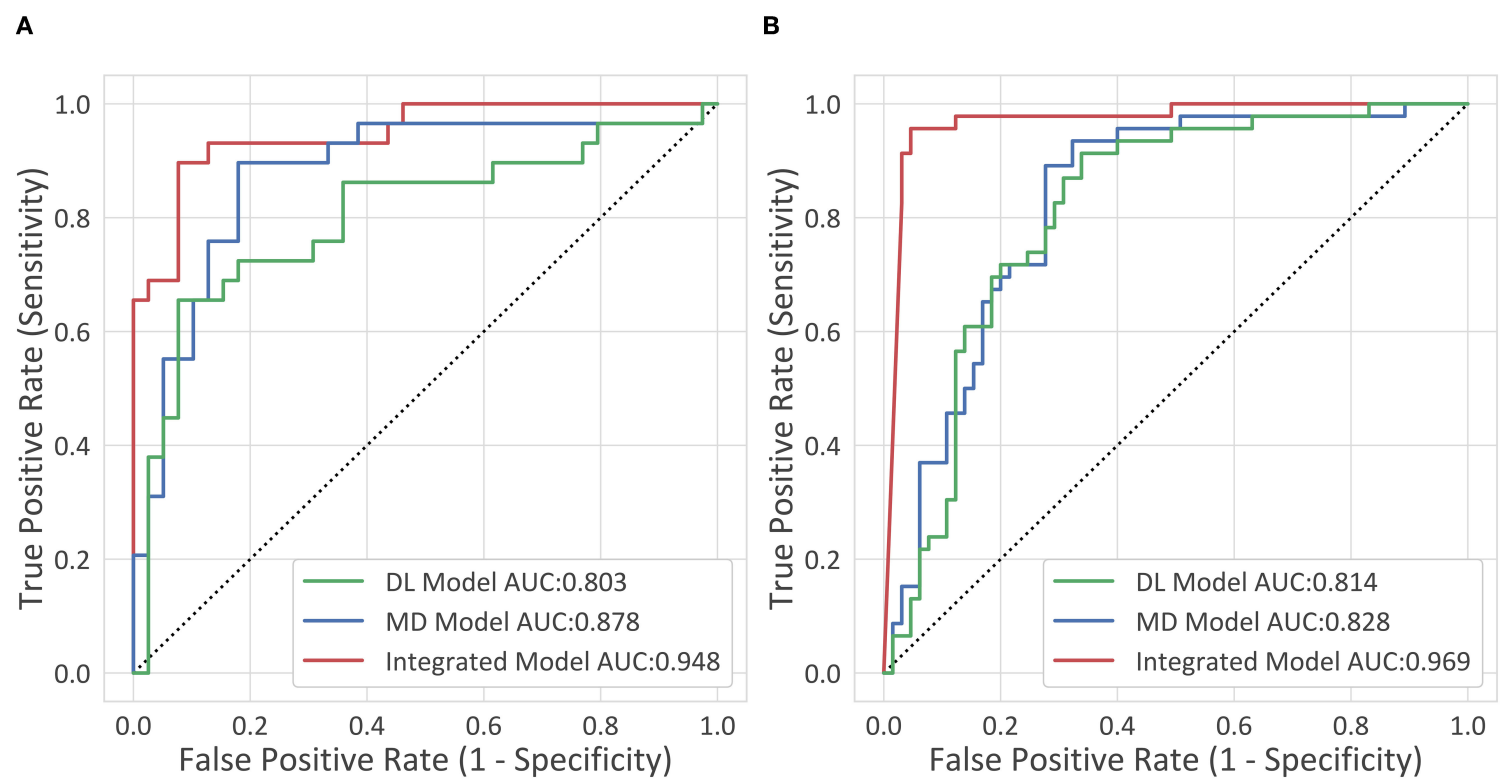
The receiver operating characteristic (ROC) curves. **(A)** The internal testing cohort. **(B)** The external testing cohort.

The diagnostic performance of each model is shown in Table 2 and the results of the receiver operating characteristic (ROC) curve analysis are shown in Figure 3.

**Table 2:** Detailed aortic dissection (AD) detection performance results of the models.

	**AUC** **(95% CI)**	**Accuracy** **(95% CI)**	**Sensitivity** **(95% CI)**	**Specificity** **(95% CI)**
**The 3D DL model**				
Training cohort	0.997 (0.993–1)	0.958 (0.922–0.979)	0.927 (0.851–0.968)	0.979 (0.935–0.995)
Cross validation result	0.84 (0.7–0.98)	0.857 (0.69–0.946)	0.857 (0.562–0.975)	0.857 (0.626–0.962)
Internal testing cohort	0.803 (0.688–0.917)	0.794 (0.675–0.879)	0.655 (0.457–0.814)	0.897 (0.748–0.967)
External testing cohort	0.814 (0.733–0.895)	0.757 (0.664–0.831)	0.891 (0.756–0.959)	0.662 (0.533–0.771)
**The deep-MD model**				
Training cohort	0.961 (0.938–0.985)	0.919 (0.879–0.948)	0.936 (0.869–0.972)	0.908 (0.85–0.946)
Cross validation result	0.953 (0.923–0.983)	0.916 (0.875–0.945)	0.936 (0.869–0.972)	0.902 (0.843–0.941)
Internal testing cohort	0.878^*^ (0.788–0.968)	0.794 (0.675–0.879)	0.690 (0.49–0.84)	0.872 (0.718–0.952)
External testing cohort	0.828 (0.751–0.906)	0.721 (0.626–0.8)	0.957 (0.84–0.992)	0.554 (0.426–0.675)
**The deep-integrated model**				
Training cohort	0.962 (0.939–0.986)	0.919 (0.879–0.948)	0.9 (0.824–0.947)	0.933 (0.879–0.964)
Cross validation result	0.956 (0.929–0.983)	0.919 (0.879–0.948)	0.909 (0.835–0.953)	0.926 (0.872–0.96)
Internal testing cohort	0.948^**^ (0.898–0.998)	0.897 (0.793–0.954)	0.862 (0.674–0.955)	0.923 (0.78–0.98)
External testing cohort	0.969^***^ (0.937–1)	0.73 (0.636–0.808)	0.978 (0.87–0.999)	0.554 (0.426–0.675)

*The p-value was calculated by the DeLong test on the internal testing cohort and the external testing cohort*.

* *p < 0.05*.

** *p < 0.01*.

*** *p < 0.001*.

The deep-integrated model built on the combination of the DL score and all the morphological characteristics achieved an AUC of 0.948 (95% CI, 0.898–0.998) in the internal testing cohort and 0.969 (95% CI, 0.937–1) in the external testing cohort. The cutoff value that maximized the Youden index was 0.33, resulting in an accuracy of 0.897 (95% CI, 0.793–0.954), a sensitivity of 0.862 (95% CI, 0.674–0.955), and a specificity of 0.923 (95% CI, 0.78–0.98) in the internal testing cohort and an accuracy of 0.73 (95% CI, 0.636–0.808), a sensitivity of 0.978 (95% CI, 0.87–0.999), and a specificity of 0.554 (95% CI, 0.426–0.675) in the external testing cohort.

**Figure 4:**
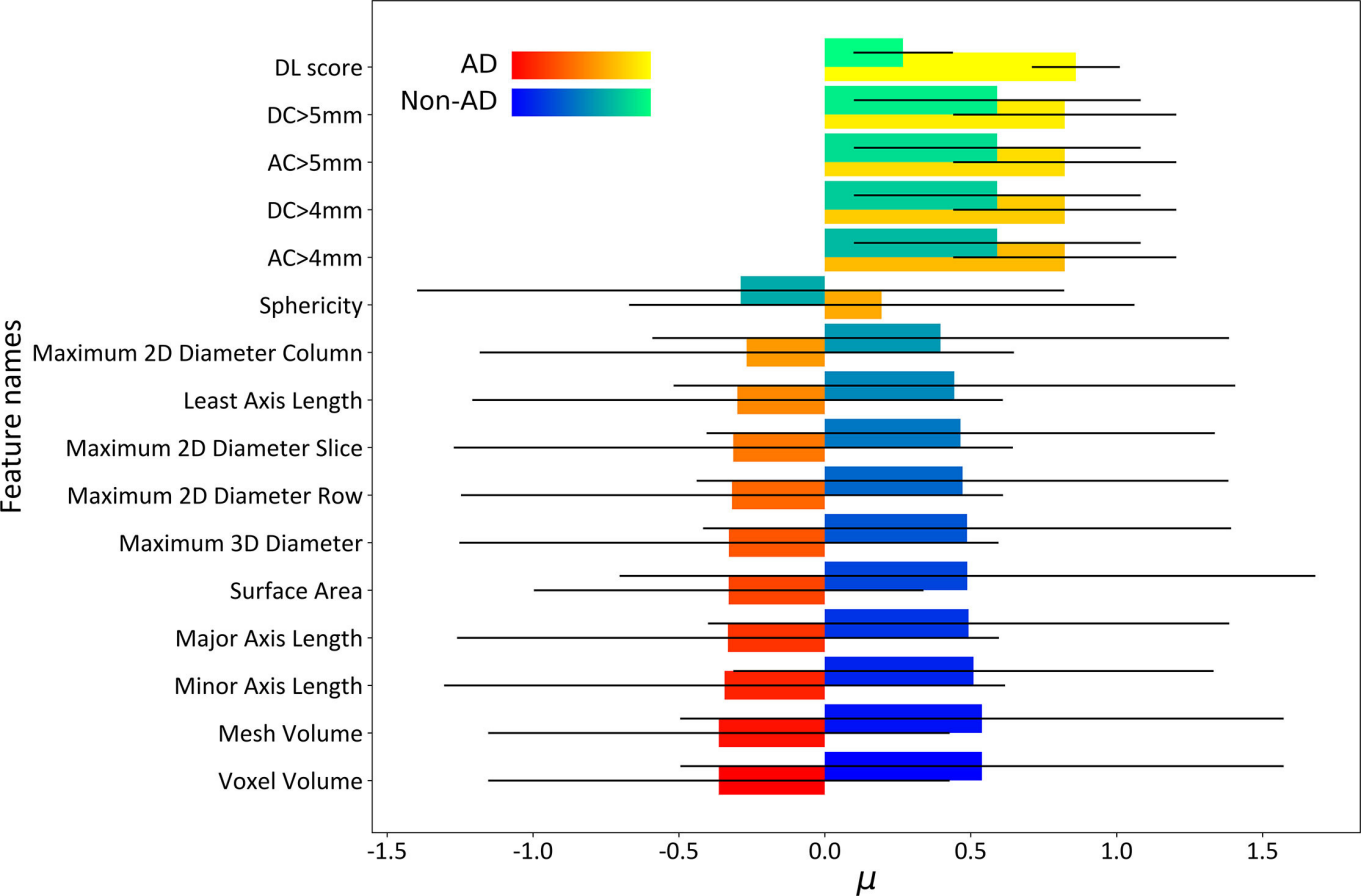
The μ and σ parameters of the deep-integrated model. The σparameter was used as an error bar. In general, patients with AD tend to have the higher DL scores and higher ascending aorta (AC) and descending aorta (DC). However, most of the general morphological features were lower in AD cases, except sphericity.

After the feature selection procedure, 16 features were used to build the deep-integrated model including the DL score, 4 maximum aortic diameter features, and 11 general morphological features. Figure 4 shows the μ and σ parameters of the trained integrated model (17) as well as the feature names. The μ parameter is the mean of each feature per class and the σ parameter is the SD of each feature per class. In general, patients with AD tend to have higher DL scores and higher AC and DC. However, most of the general morphological features were lower in AD cases, except sphericity. It should be noted that the radiomic scores were normalized by z-score normalization. The selected features and the corresponding μ and σ coefficients were given in Table 3.

**Table 3:** The list of selected features and the corresponding μ and σ coefficients of the trained deep-integrated model.

	**AD**		**Non-AD**	
**Selected features**	**μ**	**σ**	**μ**	**σ**
DL score	0.860	0.023	0.268	0.029
Least axis length	−0.300	0.827	0.444	0.927
Major axis length	−0.332	0.862	0.493	0.798
Maximum 2D diameter column	−0.268	0.838	0.397	0.977
Maximum 2D diameter row	−0.318	0.863	0.472	0.830
Maximum 2D diameter slice	−0.314	0.918	0.465	0.759
Maximum 3D diameter	−0.329	0.853	0.488	0.819
Mesh volume	−0.363	0.626	0.538	1.069
Minor axis length	−0.344	0.925	0.509	0.677
Sphericity	0.195	0.750	−0.289	1.230
Surface area	−0.330	0.446	0.489	1.421
Voxel volume	−0.363	0.626	0.538	1.069
AC > 4 mm	0.822	0.146	0.591	0.242
DC > 4 mm	0.822	0.146	0.591	0.242
AC > 5 mm	0.822	0.146	0.591	0.242
DC > 5 mm	0.822	0.146	0.591	0.242

### Comprehensive Analysis of the Performance of the Model

After training for 20 epochs [8 Garment Production Units (GPUs), Nvidia Titan Xp, ~34 h], the performance of the 3D DL model was evaluated (1 GPU, Nvidia Titan Xp, 1.2 s per CT). The 3D DL model reached an AUC of 0.803 (0.688–0.917) in the internal testing cohort and 0.814 (0.733–0.895) in the external testing cohort, which was significantly inferior to the integrated model (*p* = 0.02 and *p* < 0.001, respectively). Combined with the aortic maximum diameters, the MD model reached an AUC of 0.878 (0.788–0.968) in the internal testing cohort, which was significantly superior to the 3D DL model (*p* = 0.04). In the external testing cohort, the MD model reached an AUC of 0.828 (0.751–0.906), which was superior to the 3D DL model, but not significantly. For the comparison between the MD model and the integrated model, the AUC of the integrated model was significantly higher than that of the MD model, indicating the effectiveness of the general morphological features.

In the internal testing cohort, the deep-integrated model reached accuracies of 0.923 and 0.813 on the Stanford type A subset and the Stanford type B subset, respectively, while in the external testing cohort, they were 1.000 and 0.960. The performance on the Stanford type A subset was better than that on the Stanford type B subset, but the difference was not significant (Table 4).

**Table 4:** Accuracy comparison in the detection of AD with the Stanford type A and the Stanford type B in the two centers.

	**Internal validation cohort**				**External validation cohort**			
	**Data number**	**True positive number**	**Accuracy**	***p***	**Data number**	**True positive number**	**Accuracy**	***p***
Stanford type A	13	12	0.923	0.751	21	21	1	1.000
Stanford type B	16	13	0.813		25	24	0.96	

In the external testing cohort, the sensitivity was lower than that in the internal testing cohort. The AUC and sensitivity of the deep-integrated model were consistently determined on the subsets divided by the slice thickness. However, the specificity on the thicker subset was lower than that on the thinner subset (*p* = 0.06) (Table 5).

**Table 5:** Performance comparison in the detection of AD on different slice thickness scans in the external testing cohort.

	**AUC**	**Accuracy**	**Sensitivity**	**Specificity**
Slice thickness <8 mm (*n* = 48)	0.955	0.771	0.937	0.687
Slice thickness ≥8 mm (*n* = 63)	0.982	0.698	1.000	0.424
*p*-value	1.000	0.525	0.747	0.060

### Compared With the Radiologists Interpretation

The Fleiss's kappa coefficient among the three radiologists was 0.80 and 0.51 in the internal testing cohort and the external testing cohort, indicating substantial consistency and moderate consistency, respectively.

**Figure 5:**
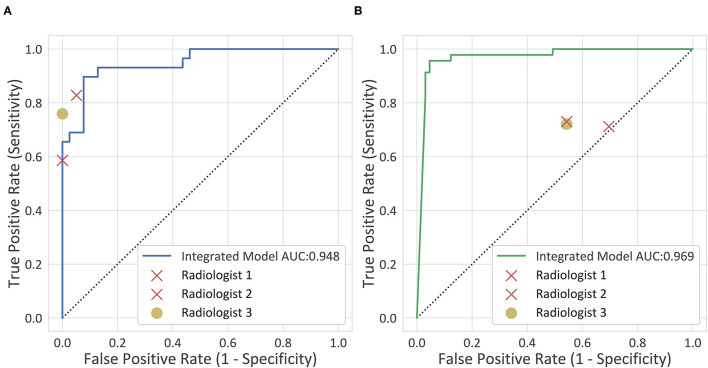
Clinical comparison. The diagnostic performance of the deep-integrated model and the three observers are shown. **(A)** The internal testing cohort. **(B)** The external testing cohort.

**Figure 6:**
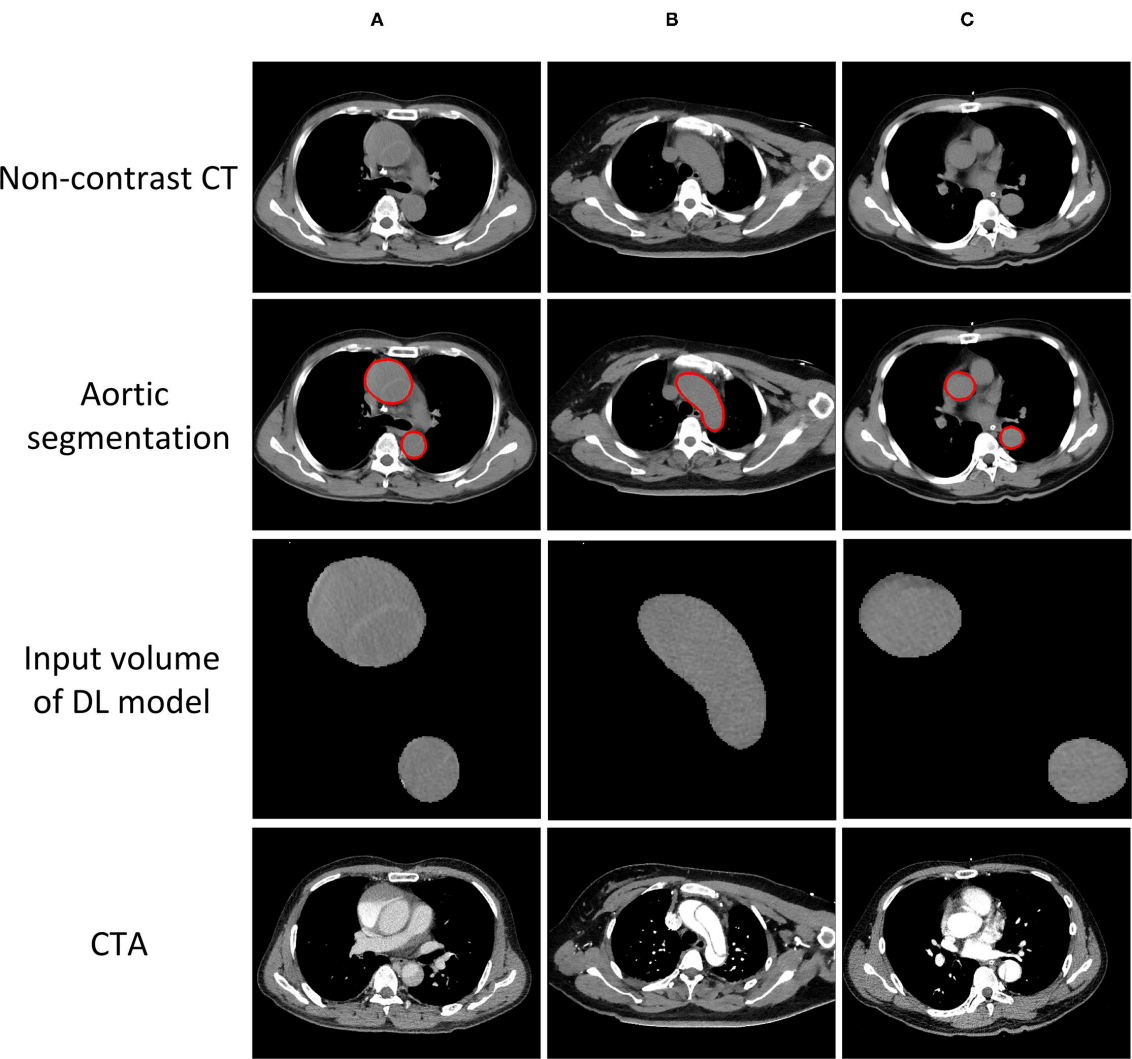
AD cases with CT images. **(A)** Successfully detected by all the radiologists and the model. **(B)** Successfully detected by our model, but neglected by one of the radiologists. **(C)** Neglected by both our model and all the radiologists.

The accuracy of the deep-integrated model was superior or equal to that of the three radiologists in both the internal and external testing cohorts, but not significantly. The sensitivity of the deep-integrated model was higher than that of all the three radiologists. It was significant between the deep-integrated model and radiologist 2 (*p* = 0.04) in the internal testing cohort and was significant between the deep-integrated model and all the three radiologists in the external validation cohort (*p* < 0.001). However, the specificity of the deep-integrated model was lower than that of all the three radiologists. No significance was found in the internal testing cohort, but it was significant in the external testing cohort (*p* < 0.001) (Table 6, Figure 5). Figure 6 shows the AD cases with CT images.

**Table 6:** Performance comparison of the model and radiologists in the detection of AD.

	**Accuracy**	**Sensitivity**	**Specificity**	**PPV**	**NPV**
	**(95% CI)**	**(95% CI)**	**(95% CI)**	**(95% CI)**	**(95% CI)**
**Internal validation cohort**					
Deep-integrated model	0.897 (0.793–0.954)	0.862 (0.674–0.955)	0.923 (0.78–0.98)	0.893 (0.706–0.972)	0.9 (0.754–0.967)
Radiologist 1	0.897 (0.793–0.954)	0.828 (0.635–0.935)	0.949 (0.814–0.991)	0.923 (0.734–0.987)	0.881 (0.736–0.955)
Radiologist 2	0.824 (0.708–0.902)	0.586^*^ (0.391–0.759)	1 (0.888–1)	1 (0.771–1)	0.765 (0.622–0.868)
Radiologist 3	0.897 (0.793–0.954)	0.759 (0.561–0.89)	1 (0.888–1)	1 (0.815–1)	0.848 (0.705–0.932)
**External validation cohort**					
Deep-integrated model	0.73 (0.636–0.808)	0.978 (0.87–0.999)	0.554 (0.426–0.675)	0.608 (0.487–0.717)	0.973 (0.842–0.999)
Radiologist 1	0.73 (0.636–0.808)	0.457^***^ (0.312–0.608)	0.923^***^ (0.822–0.971)	0.808 (0.6–0.927)	0.706^**^ (0.596–0.797)
Radiologist 2	0.712 (0.617–0.792)	0.304^***^ (0.182–0.459)	1^***^ (0.93–1)	1^*^ (0.732–1)	0.67^***^ (0.566–0.76)
Radiologist 3	0.721 (0.626–0.8)	0.457^***^ (0.312–0.608)	0.908^***^ (0.803–0.962)	0.778 (0.573–0.906)	0.702^**^ (0.591–0.795)

*The p-value was calculated by the Pearson's chi-squared test with the Yates' continuity correction*.

* *p < 0.05*.

** *p < 0.01*.

*** *p < 0.001*.

## Discussion

This study initially developed and trained a machine model that integrated the DL model and morphological characteristics of non-contrast CT images and then validated its performance at two medical centers. The results showed that the deep-integrated model was comparable to or slightly outperformed the human expert interpretation of radiologists with intermediate to high amounts of experience. This deep-integrated model could potentially support the early detection of AD based on non-contrast CT images and help to optimize the clinical workflow.

Computed tomography angiography is the best imaging modality to diagnose AD (2, 3, 18, 19), while CTA is commonly restricted in some emergency departments, especially in rural or underserved areas that lack technical and staff support (4). Making the best use of non-contrast CT scans to assist the early warning of AD in clinical practice is of great significance and has the potential to greatly improve patient outcomes. However, the poor sensitivity and high false-negative (FN) detection value reported in previous studies (9) are major concerns. The underlying causes might be related to the threshold of detecting subtle differences in grayscale images by the naked eye (8). This is supported by the observation that AD intimal rupture with relatively normal outline morphology is difficult to identify by radiologists. In addition, it is also difficult for human experts to distinguish between ruptured aortic aneurysms and unruptured aortic aneurysms on non-contrast CT images.

The DL technology has been increasingly applied to medical data analysis and CT-assisted diagnosis and has demonstrated great abilities in several issues. Studies have reported that the DL technology contributes greatly to expanding the amount of information accessible in CT images beyond human recognizability limitations. Recently, Hata et al. (12) designed the 2D DL algorithm for the detection of AD on non-contrast CT and reached an AUC of 0.940 on the internal testing set. The results of the 2D model showed comparable diagnostic performance to radiologists, which was consistent with the observation made in this study. However, their 2D algorithm did not utilize 3D spacing information and the thresholds used to generate study-based AD detection results from the 2D results could vary among different datasets. This issue might limit its application in clinical systems. The performance of the previously reported 2D model was inferior to our integrated 3D model (AUC: 0.948 on the internal testing cohort and 0.969 on the external testing cohort). In addition, the imaging data of the previous study were collected from a single center, which might lead to potential issues with reliability and reproducibility of the results.

In this study, we proposed a deep-integrated model, a Gaussian NB algorithm-based model, for the early detection of AD using non-contrast CT scans and integrated both the DL model and morphological characteristics. This model has been validated by datasets from two independent clinical centers. The aorta volume was retrieved by an aorta segmentation model and only the aorta pixel value was kept in the aorta volume. This approach reduces unrelated context noise and enables AD detection to focus on aorta detection and reduces the input size of the DL model such that the input can maintain a higher resolution. The higher resolution input increases the sensitivity of AD detection. Morphological characteristics were extracted from the aorta mask. After the DL model was built, a Gaussian NB algorithm-based model (deep-integrated model) was built on the combination of the DL score and morphological characteristics, demonstrating that the combination of morphological characteristics can strengthen the model performance. The DL model was used to capture the texture information, while the morphological features were used to capture the shape-based information. Thus, the DL score and morphological features provided complementary information.

The average sensitivity and specificity values of human expert interpretation in predicting AD on non-enhanced CT in this study were consistent with the results from previous studies and the sensitivity on the internal testing cohort was increased to 70–80% compared to a previous study result of 59–61% (9), except for radiologist 2. More predictive markers might partially contribute to the sensitivity improvement. In addition, all the participating radiologists in this study are from large academic medical centers and have experience specific to cardiovascular imaging, which may have contributed to their superior performance. It is, therefore, conceivable that general radiologists working in the community would have lower performance in the detection of AD on non-contrast CT scans. Compared to the radiologists, our model integrated the score of the DL model, which can detect the subtle textures that correlate with AD status. While the proposed AD detection model showed a significant advantage in detection sensitivity, it may be helpful for overcoming the weaknesses of human expert interpretation.

Notably, the specificity of the deep-integrated model was improved to 92.3% in the internal testing cohort, which was slightly lower than that of the radiologists and apparently higher than the corresponding results from a prior study of 85.5% (12). However, in the external testing cohort, the specificity performance decreased to 55.4%. After the exclusion of the thick scans, the specificity of the deep-integrated model increased to 68.7%. Thus, the decreasing specificity might be related to the differences in the image data and scanning parameters between the different medical centers. On one hand, higher-resolution CT can provide more diagnostic information (20, 21). Thus, thicker images may exclude some information for the detection of AD. On the other hand, there was a difference between the training cohort and external testing cohort in terms of layer thickness, which may have cause some degradation of the model performance.

The anatomic classification of AD mainly reflects the extent of the dissections and the location of the intimal tear and evaluates the degree and prognosis of lesions to guide the selection of clinical personalized treatment and operation. As indicated in this study, the detection efficiency of the deep-integrated model for AD of the Stanford type A was superior to that of the Stanford type B. This outcome could mainly be explained by the wider dissection range involved and the probability of more information and characteristics related to AD of the Stanford type A than that of the Stanford type B.

Although the CTA scan for diagnosing aortic dissection cannot be replaced by non-contrast CT in the near future and this study might not be the optimal or only approach for every patient, it is expected that the deep-integrated model could potentially be applied in the clinical setting to support clinical decision-making and improve the early detection of suspected AD in some cases. We supposed that it might help in conditions when CTA is not that convenient or timely. The significance of this technique is related to the early detection of asymptomatic patients. Furthermore, a specific group of atypical or asymptomatic patients with AD would particularly benefit from this assessment model, as it is highly likely that the diagnosis of their condition would otherwise have been missed. The model might be more helpful to community radiologists who lack specific experience or training in cardiovascular imaging and less experienced radiologists. Another possible solution is to generate synthetic CTA from non-contrast CT images (22, 23). However, this approach is hampered by the lack of a comprehensive dataset, which may lead to bias in the generated CTA and might be potentially harmful for the robustness of the model.

The main limitations of this study are as follows. First, the sample size of this study was small and relatively low accuracy results were obtained in the external testing cohort; therefore, a detailed analysis based on the subtypes of AD was not possible. The increase in specificity indicated that the slice thickness may partly explain the decline in specificity. However, the specificity was still not satisfactory, indicating that other reasons (image quality, manufacturer, etc.) contributed to the decline in specificity, but these factors were not analyzed. In addition, this was a retrospective study and detailed information about initial symptoms and the purpose for CT examination of the enrolled patients were incomplete. However, the prevalence of asymptomatic and unsuspected patients with AD might be significant for the clinical use of this model. Furthermore, potential challenges (e.g., inconsistency in image quality, contrast and imaging protocols from different centers) might be necessary for translating this method into a clinical tool. To validate the clinical potential of the model, multicenter prospective trials with a range of CT examination types will be needed to further investigate the reliability and reproducibility of our results.

## Conclusion

The deep-integrated model, an integrated matching learning model, was comparable to or slightly outperformed the human expert interpretation of radiologists with intermediate to high amounts of experience in detecting AD on non-contrast CT images. This model might contribute to the improvement in early disease detection and downstream clinical decision optimization for patients at risk for AD.

## Data Availability Statement

The original contributions presented in the study are included in the article/Supplementary Material, further inquiries can be directed to the corresponding authors.

## Ethics Statement

Written informed consent was obtained from the individual(s) for the publication of any potentially identifiable images or data included in this article.

## Author Contributions

YY, YG, YW, and ZJ designed the study. YY, YG, and XL acquired and collected the data in clinical studies. YY, YG, YW, LM, CW, and DJ analyzed and interpreted the data. LM performed the statistical analysis. YY and LM drafted the manuscript. YL, JP, JL, and SL revised the manuscript critically for important intellectual content. YW and ZJ are the guarantors of the integrity of the entire study. All the results were checked by CW and X-LL. All authors finally approved for submitting the manuscript.

## Funding

This work was supported by CAMS Innovation Fund for Medical Sciences (CIFMS) (Grant No. 2020-I2M-C-T-B-034), the Major International (Regional) Joint Research Project of National Natural Science Foundation of China (Grant No. 82020108018, 2021), the Beijing Natural Science Foundation (Grant No. Z210013), the National Natural Science Foundation of China (Grant No. 81873891), and the China Postdoctoral Science Foundation (2020T130071).

## Conflict of Interest

LM, CW, DJ, and X-LL were employed by Deepwise AI lab. The remaining authors declare that the research was conducted in the absence of any commercial or financial relationships that could be construed as a potential conflict of interest.

## Publisher's Note

All claims expressed in this article are solely those of the authors and do not necessarily represent those of their affiliated organizations, or those of the publisher, the editors and the reviewers. Any product that may be evaluated in this article, or claim that may be made by its manufacturer, is not guaranteed or endorsed by the publisher.
