# Supplementary material for: Advanced Warning of Aortic Dissection on Non-Contrast CT: The Combination of Deep Learning and Morphological Characteristics
Source: Front Cardiovasc Med. 2022 Jan 5;8:762958. doi: 10.3389/fcvm.2021.762958 (PMC8767113; doi:10.3389/fcvm.2021.762958)
Supplement: Supplementary file 1 [file Data_Sheet_1.docx]

**S1. Patient population**

Between July 2014 and April 2020, there were 5885 consecutive patients underwent CTA scans from Peking Union Medical College Hospital (PUMCH), Beijing, China. The presence of AD was confirmed by the CTA interpretation results, and 191 patients were diagnosed with Aortic Dissection (AD) and underwent CTA scans. The inclusion criteria were as follows: (a) patients diagnosed with AD and underwent CTA scans. The exclusion criteria were as follows: (a) patients with aortic endovascular treatment (n=15). (b) patients without non-enhanced scan (n=10). (c) patients with AD occurred merely in abdominal cavity (n=21). (d) patients without CT in thoracic cavity (n=6). A total of 202 patients diagnosed without AD and underwent CTA scans from the same period were approximately propensity-matched from the remaining 5694 non-AD patients, considering 2 variables (Age, gender). Thus, 341 patients were enrolled from PUMCH (139 AD patients and 202 non-AD patients) and were randomly divided into the training cohort (70%, 238 patients with 96 AD patients and 142 non-AD patients), the validation cohort (10%, 35 patients with 14 AD patients and 21 non-AD patients) and the internal testing cohort (20%, 68 patients with 29 AD patients and 39 non-AD patients).

From another independent medical center, Shenzhen Second People's Hospital (SSPH), Shenzhen, China, 2273 consecutive patients underwent CTA scans between July 2017 and June 2020. Among them, 70 patients were diagnosed with AD. After the same inclusion and exclusion criteria, 46 AD patients were enrolled, and 65 patients were propensity-matched, then the external testing cohort was constructed.

**S2. Aorta Segmentation**

The aorta segmentation algorithm was a 2.5D UNet-based deep learning model. The model's input was the combination of the target slice with its upper and lower slices. Then the generated aorta masks of all slices were assembled to form a 3-dimensional aorta mask. To reduce the false-positive rate, we implemented a connected component analysis algorithm as post-processing to remove outliers. The aorta segmentation algorithm was trained and validated on the in-house dataset of 236 non-contrast CT series, and the generated 3-dimensional aorta mask reached a Dice score of 92%.

**S3. morphological characters**

The aortic maximum diameters included the maximum diameters of ascending aorta and descending aorta. The calculation was based on the aorta segmentation inferred by deep learning model, and the method for differentiation of the ascending aorta and descending aorta was elaborate below. After the discrimination of the ascending aorta and descending aorta, we calculated the diameters of these contours in each slice and get the maximum diameters of ascending aorta and descending aorta. The aortic maximum diameters were binarized by the threshold of 4cm and 5cm to form 4 aortic maximum diameter features, i.e., AC>4cm, AC>5cm, DC>4cm, and DC>5cm.

The discrimination of the ascending aorta and descending aorta can be divided into 3 steps. (1) Finding of the aorta arch. We use the segmentation to get the 3d skeleton of the aorta, i.e. the center line of the segmentation. Then we calculate the sum of the skeleton points every three layers and locate the aorta arch which has the maximum value. The aorta arch located at the center of the three layers. (2) Finding the range of the ascending aorta and descending aorta. There is only one segmentation contour in each slice of the aorta arch and we can find the end of the arch when there two contours begin to appear. (3) The discrimination of the ascending aorta and descending aorta. The reference point was calculated by averaging the skeleton points of the aorta arch. Then for each slice, the center point of each contour was calculated and compared with the reference point, and the contour was appointed to the ascending aorta or descending aorta depended on anatomical position (If the CT is processed to be head above foot down, the center points of ascending aorta is in the left direction of the reference point and that of descend aorta is in the right direction).

For the general morphologic features, we use the PyRadiomic(v3.0) to extract the shape based features. Then the general morphologic features were normalized by z-score normalization. The features were listed below. For formulas of each feature, please refer to the PyRadiomics official website: <https://pyradiomics.readthedocs.io/en/latest/features.html> .

Shape features describe the morphological property of the VOI were generated from the aorta mask. $N_{v}$ represents the number of voxels included in the VOI. $N_{f}$ represents the number of faces (triangles) defining the Mesh.

**Table S1.** Descriptions of all features for shape-based characteristics.

| **No.** | **Feature names** | **Descriptions** |
| --- | --- | --- |
| 1 | Elongation | Elongation shows the relationship between the two largest principal components in the VOI shape. |
| 2 | Flatness | Flatness shows the relationship between the largest and smallest principal components in the VOI shape. |
| 3 | Least Axis Length | This feature yields the smallest axis length of the VOI-enclosing ellipsoid and is calculated using the largest principal component  $\lambda_{\mathrm{least}}$. |
| 4 | Major Axis Length | This feature yields the largest axis length of the VOI-enclosing ellipsoid and is calculated using the largest principal component  $\lambda_{major}$. |
| 5 | Maximum 2D Diameter Column | Maximum 2D diameter (column) is defined as the largest pairwise Euclidean distance between tumour surface mesh vertices in the row-slice (usually the coronal) plane. |
| 6 | Maximum 2D Diameter Row | Maximum 2D diameter (row) is defined as the largest pairwise Euclidean distance between tumour surface mesh vertices in the column-slice (usually the sagittal) plane. |
| 7 | Maximum 2D Diameter Slice | Maximum 2D diameter (slice) is defined as the largest pairwise Euclidean distance between tumour surface mesh vertices in the row-column (generally the axial) plane. |
| 8 | Maximum 3D Diameter | Maximum 3D diameter is defined as the largest pairwise Euclidean distance between tumour surface mesh vertices. |
| 9 | Mesh Volume | The volume of the VOI (V) is calculated from the triangle mesh of the VOI. For each face ii in the mesh, defined by points $a_{i}$,$b_{i}$, and $c_{i}$, the (signed) volume $V_{f}$ of the tetrahedron defined by that face and the origin of the image (O) is calculated. The sign of the volume is determined by the sign of the normal, which must be consistently defined as either facing outward or inward of the VOI. |
| 10 | Minor Axis Length | This feature yields the second-largest axis length of the VOI-enclosing ellipsoid and is calculated using the largest principal component $\lambda_{\mathrm{minor}}$. |
| 11 | Sphericity | Sphericity is a measure of the roundness of the shape of the tumour region relative to a sphere. It is a dimensionless measure, independent of scale and orientation. The value range is 0$<$sphericity$\leq$1, where a value of 1 indicates a perfect sphere (a sphere has the smallest possible surface area for a given volume, compared to other solids). |
| 12 | Surface Area | To calculate the surface area, first the surface area Ai of each triangle in the mesh is calculated. The total surface area is then obtained by taking the sum of all calculated sub-areas. |
| 13 | Surface Volume Ratio | A lower value indicates a more compact (sphere-like) shape. This feature is not dimensionless, and is therefore (partly) dependent on the volume of the VOI. |
| 14 | Voxel Volume | The volume of the VOI is approximated by multiplying the number of voxels in the VOI by the volume of a single voxel. This is a less precise approximation of the volume and is not used in subsequent features. This feature does not make use of the mesh and is not used in calculation of other shape features. |
